# Seabuckthorn polysaccharide combined with *astragalus* polysaccharide ameliorate alcoholic fatty liver by regulating intestinal flora

**DOI:** 10.3389/fendo.2022.1018557

**Published:** 2022-09-29

**Authors:** Jiayue Liu, Lingzhou Kong, Mengting Shao, Changhai Sun, Changxu Li, Yanyan Wang, Xue Chai, Yuliang Wang, Yu Zhang, Xiaoliang Li, Hong Zhao

**Affiliations:** ^1^ College of Pharmacy, Heilongjiang Provincial Key Laboratory of New Drug Development and Pharmacotoxicological Evaluation, Jiamusi University, Jiamusi, China; ^2^ Key Laboratory of Tropical Translational Medicine of Ministry of Education, Hainan Provincial Key Laboratory for Research and Development of Tropical Herbs, Haikou Key Laboratory of Li Nationality Medicine, School of Pharmacy, Hainan Medical University, Haikou, China

**Keywords:** Seabuckthorn, *Astragalus*, polysaccharide, alcoholic fatty liver, intestinal flora

## Abstract

**Background:**

At present, the incidence of alcoholic fatty liver disease (AFLD) is increasing year by year, and numerous studies have confirmed that liver diseases are closely related to intestinal flora. Seabuckthorn and *Astragalus membranaceus*, as traditional Chinese medicine (TCM) with the homology of medicine and food, have good liver protection, and their polysaccharides can regulate the intestinal flora. Here, we studied the effects of HRP, APS and the combination of the two polysaccharides on the intestinal flora of AFLD mice, which provided scientific basis for the treatment of AFLD with the two polysaccharides.

**Materials and methods:**

Thirty Kunming (KM) mice were randomly divided into the control group (Con), the model group (Mod), the HRP treatment group (HRP), the APS treatment group (APS), and HRP+APS treatment group (HRP+APS), with six mice in each group. The AFLD model was constructed by continuous intragastric administration of 42% vol Niulanshan ethanol solution for 28 days, and the mice in each polysaccharide group were given corresponding drugs. The levels of AST, ALT, TC and TG in serum of mice were measured. 16S rRNA amplicon sequencing technique was used to determine the diversity and richness of intestinal flora, and the relative abundance of intestinal flora at phylum level and genus level of the mice in each group.

**Results:**

HRP, APS and HRP+APS could reduce the serum levels of AST, ALT, TC and TG in mice. In addition, HRP, APS and HRP + APS restored the diversity, relative abundance and community structure of intestinal mucosa bacteria in AFLD mice to a certain extent. Specifically, HRP, APS and HRP+APS remarkably decreased the ratio of *Firmicutes* to *Bacteroidetes*, and ultimately increased the abundance of beneficial bacteria and reduced the abundance of pathogenic bacteria.

**Conclusion:**

HRP, APS, and HRP+APS can improve the intestinal microecology of AFLD model mice, alleviate liver injury, and maintain normal intestinal function in different degrees.

## Introduction

With the development of economy and the living standard, the culture of drinking is prevalent, and excessive drinking is increasingly the norm for many people (1). According to the World Health Organization, more than 3 million alcohol-related deaths occur globally each year, accounting for 5.3% of all deaths (2). The liver is the main place for the oxidative metabolism of alcohol, and long-term alcoholism will lead to a series of liver diseases. Alcoholic liver disease is one of the common diseases, which usually manifests as AFLD in the early stage (3, 4). Luckily, AFLD can be reversed, but without timely intervention, it will develop into more serious liver diseases, such as alcoholic hepatitis, liver fibrosis, cirrhosis, and even deteriorate into liver cancer (5). At present, the treatment of AFLD mainly includes abstinence, nutritional support and drug therapy (6). The drug treatment mainly includes metadoxine, glucocorticoids, glycyrrhizic acid preparation and other drugs, which are not suitable for long-term use and are not specific drugs for AFLD (7, 8). Therefore, the development of high-efficiency and low-toxicity therapeutic drugs for AFLD has become an urgent clinical need.

Long-term excessive drinking leads to disturbances in the composition and distribution of intestinal microbiota, the decrease of beneficial bacteria and the increase of harmful bacteria, which in turn increases the permeability of the intestinal wall and induces endotoxemia (9). Lipopolysaccharide (LPS) from Gram-negative bacteria in the gut enters the peripheral circulation through the damaged intestinal mucosal barrier and reaches the liver tissue through the portal vein blood (10). LPS overactivates Kuffer cells, which in turn induces the occurrence of inflammatory reactions and the release of inflammatory factors, ultimately leading to liver damage (11). Many studies (12, 13) have shown that intestinal dysbacteriosis and excessive expression of inflammatory factors play an important role in the occurrence and development of AFLD. TCM and its active ingredients are mostly orally entered into the body, while the active ingredients in drugs interact with intestinal flora after entering the gastrointestinal tract and exert therapeutic effects by affecting the intestinal microbiota (14–16).

Seabuckthorn, as a classic TCM for promoting blood circulation and dispersing blood stasis, can be combined with *Astragalus membranaceus* to strengthen spleen and Qi, nourish stomach and Yin. *Hippophae rhamnoides* polysaccharide (HRP), as one of the main active components of *Hippophae rhamnoides*, has been proved to have good liver protection (17). At the same time, *astragalus* polysaccharide (APS) was found to have significant anti-inflammatory, liver protection, anti-oxidation and immune-enhancing effects, and can significantly improve liver damage, which has potential therapeutic effects on AFLD (18, 19). These two polysaccharides have different degrees of liver protection. Whether the combination of the two produces can produce synergistic effects and whether the mechanism is related to the regulation of intestinal flora are the key issues to be solved.

In the present study, HRP and APS were used in an equal mass ratio to compare the therapeutic effects of HRP, APS and HRP+APS on AFLD mice. To evaluate its impact on the intestinal flora structure in AFLD mice, the 16S rRNA coding region of the mice intestinal contents were amplified and annotated by the NovaSeq 6000 system. This study laid an experimental foundation for solving the problem of limited activity of single polysaccharide and provided a new idea for the prevention and treatment of AFLD in the future.

## Materials and methods

### Animals and reagents

A total of 30 male Kunming mice of SPF grade, weighing 18-22 g, were purchased from Changchun Yisi Experimental Animal Technology Co., Ltd. (SCXK (Ji)-2018-0007). All experimental procedures involving animals were approved by Animal Ethics Committee of the Animal Experimental Center of Jiamusi University.

42% vol liquor of Niulanshan was purchased from Niulanshan distillery of Beijing Shunxin Agricultural Co., Ltd. Alanine aminotransferase (ALT), aspartate aminotransferase (AST), triglyceride (TG) and total cholesterol (TC) kits were obtained from Nanjing Jiancheng Bioengineering Institute (Nanjing, China).

### Preparation of polysaccharides

Seabuckthorn fruit was purchased from Jiamusi Minsheng pharmacy (batch number: 180312); *Astragalus* was purchased from Tongrentang pharmacy, Jiamusi City, Heilongjiang Province (batch number 170801).

After degreasing, sea buckthorn fruit was extracted three times with distilled water at the ratio of 1:23 g/ml at 90° for 3 h each time, and HRP with a polysaccharide content of 64.33% was obtained after further deproteinization. Similarly, the defatted *astragalus* was extracted 3 times with distilled water at 95°C with a ratio of 1:20 g/ml, each time for 3 h, and after deproteinization, APS with polysaccharide content of 62.73% was obtained.

### Modeling and treatment

**Figure 1:**
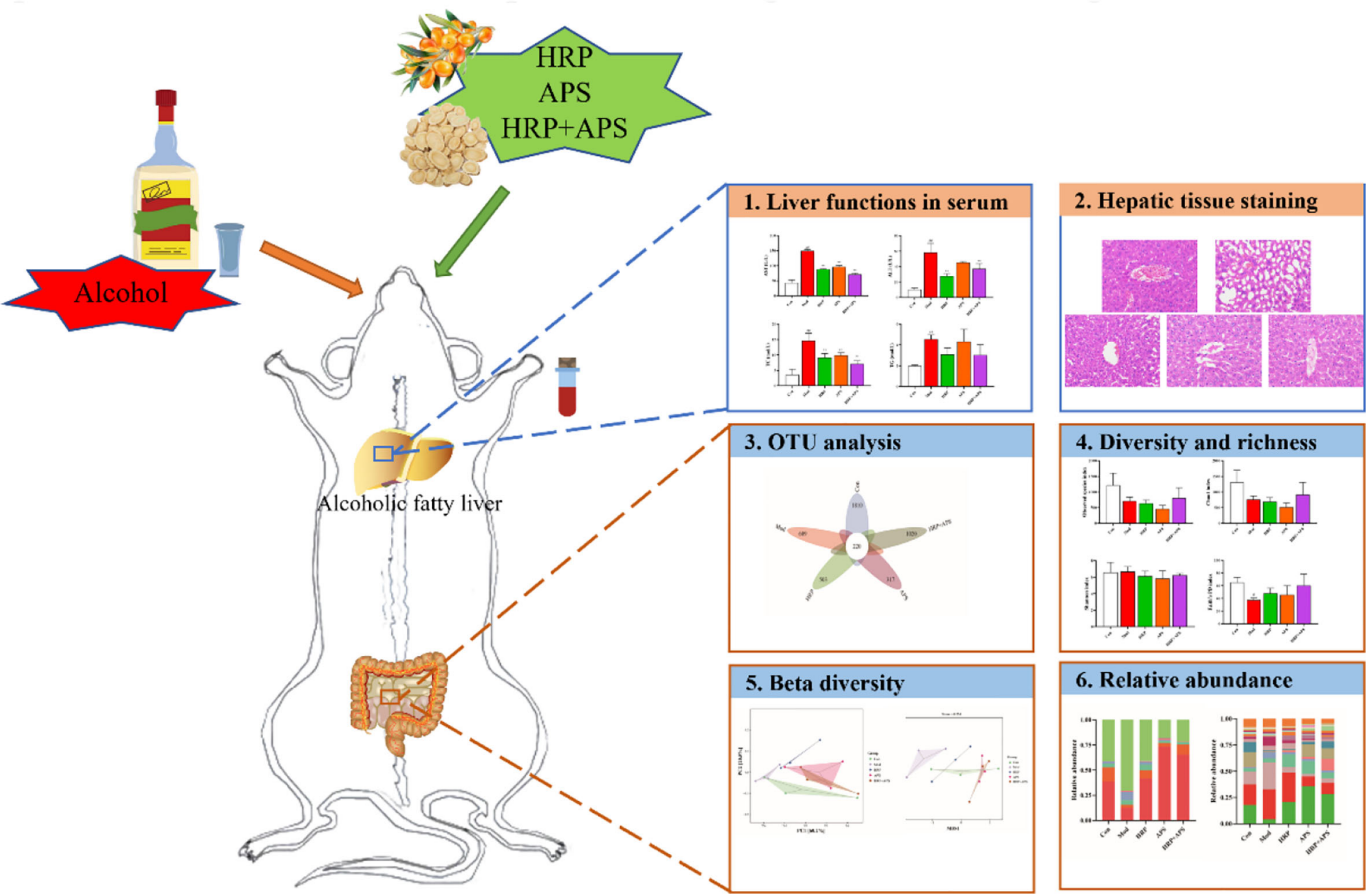
Flowchart of the experimental design.

After a week of acclimation, 30 male KM mice were divided into control group (Con), model group (Mod), HRP treatment group (HRP), APS treatment group (APS), and HRP+APS treatment group (HRP+APS) according to the random number table method, with 6 mice in each group. Except for the Con group, the mice in the other groups were orally administered 14 mL/kg 42% vol liquor of Niulanshan every day. After 6 h, mice in HRP and APS groups were given 22 mg/kg corresponding polysaccharide solution, and mice in HRP+APS treatment group were given 22 mg/kg mixed solution of HRP and APS. Con and mod groups were given the same amount of normal saline for 28 days. The body weight of mice in each group was recorded every 3 days during the experiment. Flowchart of the experimental design was shown in **Figure 1**.

### Hepatic tissue staining

After the experiment, liver tissues of mice were removed, fixed immediately in 10% neutral buffered formalin for 24h, and then embedded in paraffin. The liver tissues embedded in paraffin were cut into 5mm thick sections and stained with hematoxylin and eosin (H&E). The steatosis of liver tissue was examined under a light microscope at 400 magnifications.

### Serum biochemical analysis

Serum samples were isolated from eye blood of mice by centrifugation at 3000 rpm for 10 minutes at 4°. The level of AST, ALT, TC and TG in serum of mice were determined by automatic blood biochemical analyzer.

### 16S rRNA amplicon sequencing analyses

According to the instructions of the DNA kit, DNA was extracted from the intestinal contents of mice, quantified by Nanodrop, and the quality of DNA extraction was detected by 1.2% agarose gel electrophoresis. PCR amplification and sequencing were designed according to the V3-V4 region of 16S rRNA. The microbiota of intestinal contents of mice were sequenced using NovaSeq 6000 platform of Illumina, and the results were annotated with Green Genes 13.8 database. Sequencing was completed by Wuhan Frasergen Genomic Medicine Co., Ltd (Wuhan, China).

### Bioinformatics and statistical analysis

Non-repetitive sequences were clustered by OTU with 97% similarity using QIME2 software and compared with the SILVA database to obtain the species classification information corresponding to each OTU. Alpha diversity analysis including Chao1, ACE, Simpson and Shannon indices was calculated and analyzed using R 4.1.2. The structural variation of microbial communities across samples was investigated by the Beta diversity, including Principle component analysis (PCA) of UniFrac distance metrics and nonmetric multidimensional scaling (NMDS) (19).

SPSS 26.0 statistical software was used for data analysis. Differences between multiple groups were compared using Kruskal-Wallis and one-way ANOVA analysis, and differences between two groups were compared using T-test. The results were expressed as mean ± SD, and *P*<0.05 was considered statistically significant. Graphs were performed using R 4.2 and GraphPad Prism 8 software.

## Results

### Effects of the combination of HRP and APS on general characteristics and liver histopathological changes in AFLD mice

**Figure 2:**
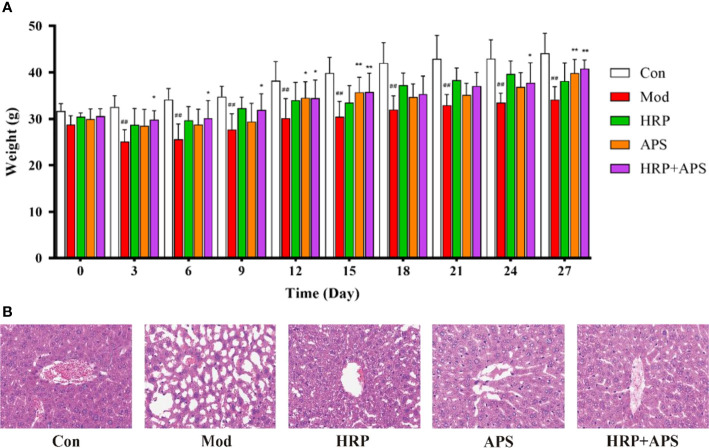
The effects of HRP, APS and the combination of HRP and APS on the body weight and histopathological change of liver in AFLD mice. **(A)** Weight changes of mice during the experiment. **(B)** Hematoxylin-eosin **(H&E)** staining of hepatocytes (Original magnification, ×400, n = 3). Data are presented as mean ± SD (n = 6). ^##^ *P* < 0.01 vs. Con; ^*^ *P* < 0.05 and ^**^ *P* < 0.01 vs. Mod.

At the beginning of the experiment, all mice exhibited normal food intake, natural spirit, smooth shiny hair, and dry black fecal pellets that did not stick to hands when squeezed. As shown in **Figure 2A**, on the third day after the administration of Chinese Baijiu, the weight of mice in the model group decreased significantly (*P*<0.01), the food intake reduced, and the hair lost luster, while no obvious abnormalities were found in other groups. On the 27th day of the experiment, compared with the model group, the weight of mice in HRP administration group increased slightly (*P*>0.05), and the weight of mice in APS and HRP+APS groups increased remarkably (*P*<0.01). Meanwhile, after the polysaccharide intervention, the hair of mice in each group became thick and shiny, and the food intake and urine volume were normal.

As shown in **Figure 2B**, the liver tissue structure of mice in the blank group was complete, and the nuclei were round and orderly arranged. By contrast, chronic ethanol exposure caused disordered arrangement of hepatocytes, increased tissue vacuoles and fat accumulation in mice. After polysaccharides intervention, the abnormal structure of liver tissue of mice in each group was improved to varying degrees. Among them, the improvement effect of HRP+APS group was more significant, and some cells in the liver tissue of HRP and APS groups were still in disorder and fat accumulation. These results showed that the combination of HRP and APS could improve the weight loss and liver injury of AFLD mice.

### Analysis of serum biochemical indexes of mice

**Figure 3:**
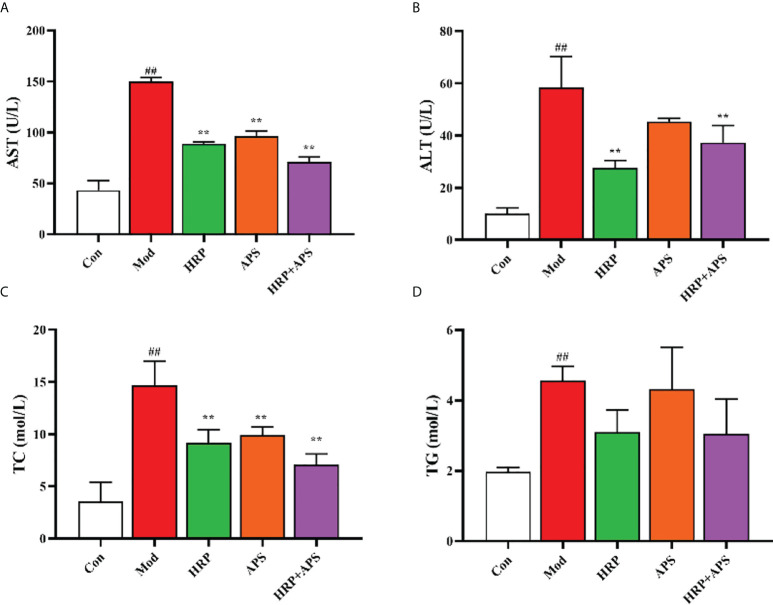
The effects of HRP, APS and the combination of HRP and APS on serum biochemical indexes of AFLD mice. **(A)** Serum AST. **(B)** Serum ALT. **(C)** Serum TC. **(D)** Serum TG. Data are presented as mean ± SD (n = 6). ^##^ *P <* 0.01 vs. Con; ^**^ *P <* 0.01 vs. Mod.

As shown in **Figure 3**, compared with the Con group, the contents of AST, ALT, TC and TG in the serum of mice in the model group were all significantly increased (*P <*0.01). Compared with the model group, AST, ALT and TC in serum of mice in HRP group were remarkably decreased (*P*<0.01), AST and TC in serum of mice in APS group were dramatically reduced (*P*< 0.01), and AST, ALT and TC in HRP + APS group were significantly decreased (*P*<0.01). After HRP and APS intervention, the serum TG content of mice decreased, but there was no significant difference. The above results implied that HRP, APS and HRP+APS had the regulating serum biochemical indexes of mice in AFLD mice.

### Effects of the combination of HRP and APS on bacterial OTU number and alpha diversity in the intestinal mucosa of AFLD mice

**Figure 4:**
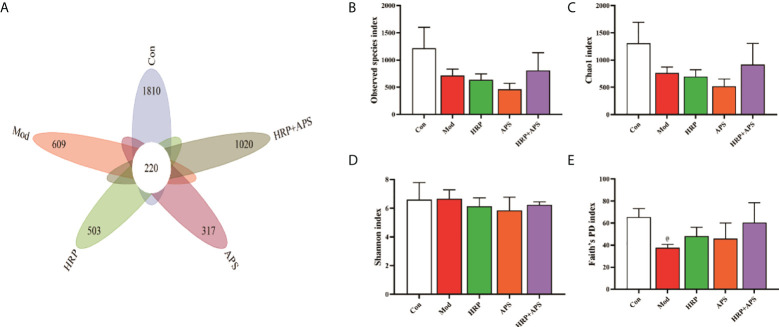
Effects of HRP, APS and the combination of HRP and APS on the number of OTUs and alpha diversity in mice intestinal mucosal bacteria. **(A)** The number of observed OTUs. **(B)** Observed species index. **(C)** Chao1 index. **(D)** Simpson index. **(E)** Faith’s PD index. ^#^ *P < *0.05 vs. Con.

The analysis of OTUs are shown in **Figure 4A**, there were 220 OTUs in the five experimental groups, and the number of OTUs in blank group, model group, HRP group, APS group, and HRP+APS group were 1810, 609, 503, 317, and 1020, respectively. Compared with the blank group, the number of OTUs in the model group was significantly decreased, indicating that the intestinal flora of mice was disordered after drinking. After the intervention of different polysaccharides, the number of OTUs in the intestinal tract of mice increased, and the HRP+APS group was the closest to the blank group.

Alpha diversity refers to the diversity within a specific area or ecosystem, and is a comprehensive indicator reflecting richness and evenness. Observed species and Chao1 indices are used to evaluate richness, and the larger its values, the more abundant the total number of species in the environment. As two other indicators for assessing diversity, Shannon and Faith’s PD index, the higher the value, the higher the diversity of species in the environment. As can be seen from **Figures 4B-E**, compared with the blank group, the Observed species and Chao1 in the intestinal flora of AFLD mice were decreased (*P*>0.05) and the Faith’s PD was significantly decreased (*P*<0.05), which indicated that alcohol caused certain damage to the diversity and richness of the intestinal flora in mice. After the polysaccharide intervention, there was no significant difference in Observed species, Chao1, Shannon and Faith’s PD index in the intestinal flora of mice in the HRP and APS groups. But, HRP + APS could increase the observed species, Chao1 and Faith’s PD index in the intestinal flora of AFLD mice (*P*>0.05), indicating that HRP + APS could restore the richness and diversity of the intestinal flora of AFLD mice.

### Effects of the combination of HRP and APS on intestinal beta diversity in AFLD mice

**Figure 5:**
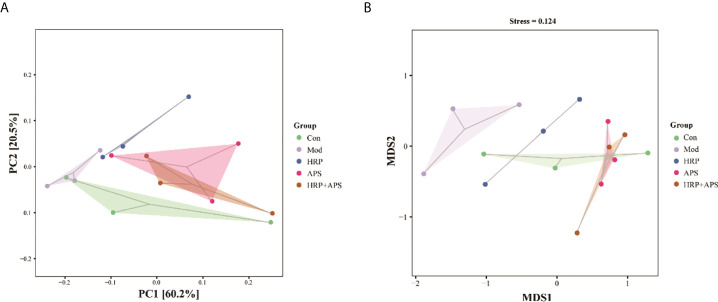
Effect of HRP, APS and the combination of HRP and APS on the beta diversity of mice intestinal mucosal bacteria. **(A)** PCA and **(B)** NMDS.

Beta diversity, also known as between-habitat diversity, is used to study the species diversity relationship between communities, including unconstrained ordination, such as PCoA and NMDS. The PCA plot (**Figure 5A**) and NMDS plot (**Figure 5B**) showed that the gut microbiota structure of the model group was remarkably different from that of the blank group, which indicated that alcohol could alter the structure of the mice gut microbiota. As expected, each administration group was relatively concentrated and had a high similarity in community structure with the blank group after HRP, APS and HRP+APS treatment. Beta diversity analysis indicated that the structure of intestinal mucosal bacteria could be repaired and restored to normal state by the combined application of HRP and APS.

### Effects of the combination of HRP and APS on relative abundance of intestinal flora in AFLD mice

**Figure 6:**
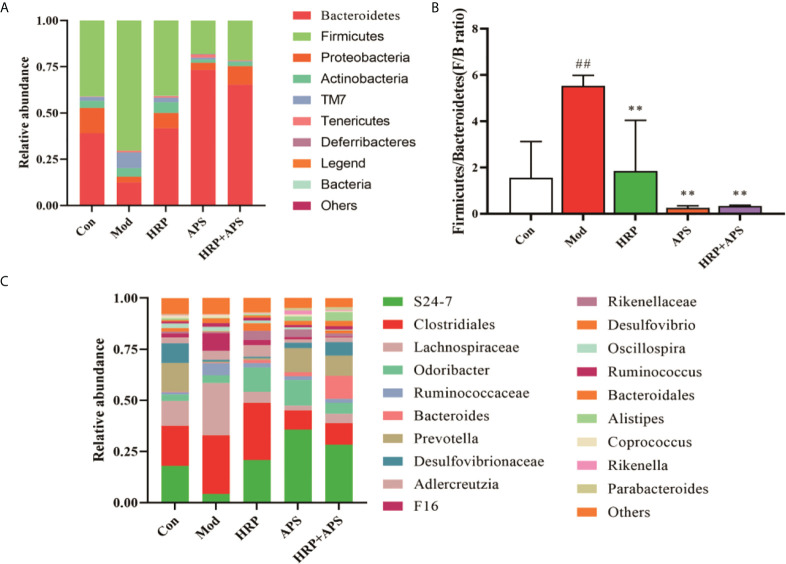
Effect of HRP, APS and the combination of HRP and APS on relative abundance of intestinal flora in AFLD mice. **(A)** Relative abundance at the phylum level. **(B)** Histogram of Firmicutes/Bacteroidetes ratio. **(C)** Relative abundance at the genus level. ^##^P < 0.01 vs. Con; **P < 0.01 vs. Mod.

The relative abundance of intestinal mucosal bacteria at the phylum level in mice is shown in **Figure 6A**, in which *Firmicutes* and *Bacteroidetes* are the dominant phyla. Compared with the blank group, the relative abundance of *Firmicutes* in the intestinal flora of the model group was significantly increased (*P*<0.01), and the relative abundance of *Bacteroidetes* was dramatically decreased (*P*<0.01). These trends were remarkably reversed after the intervention of all polysaccharide groups. As shown in **Figure 6B**, the F/B ratio of the model group was significantly increased (*P*<0.01), while the F/B ratio was significantly decreased after HRP, APS and HRP+APS treatment (*P <*0.01).

The relative abundance of intestinal microbiota in mice at the genus level are shown in **Figure 6C**, in which *S24-7*, *Clostridiales*, and *Lachnospiraceae* are the dominant genera. Compared with the blank group, the relative abundance of *S24-7* in the intestinal microbiota of mice in the model group was decreased, while the relative abundance of *Clostridiales* and *Lachnospiraceae* was increased, but there was no significant difference (*P*>0.05). After polysaccharide intervention, the relative abundance of *S24-7* in intestinal microbiota of mice in APS group and HRP+APS group was significantly increased compared with that in model group (*P*<0.01), and the relative abundance of *Clostridiales* and *Lachnospiraceae* was decreased in HRP, APS and HRP+APS groups (*P*>0.05).

### Correlation between gut microbiota and AFLD

**Figure 7:**
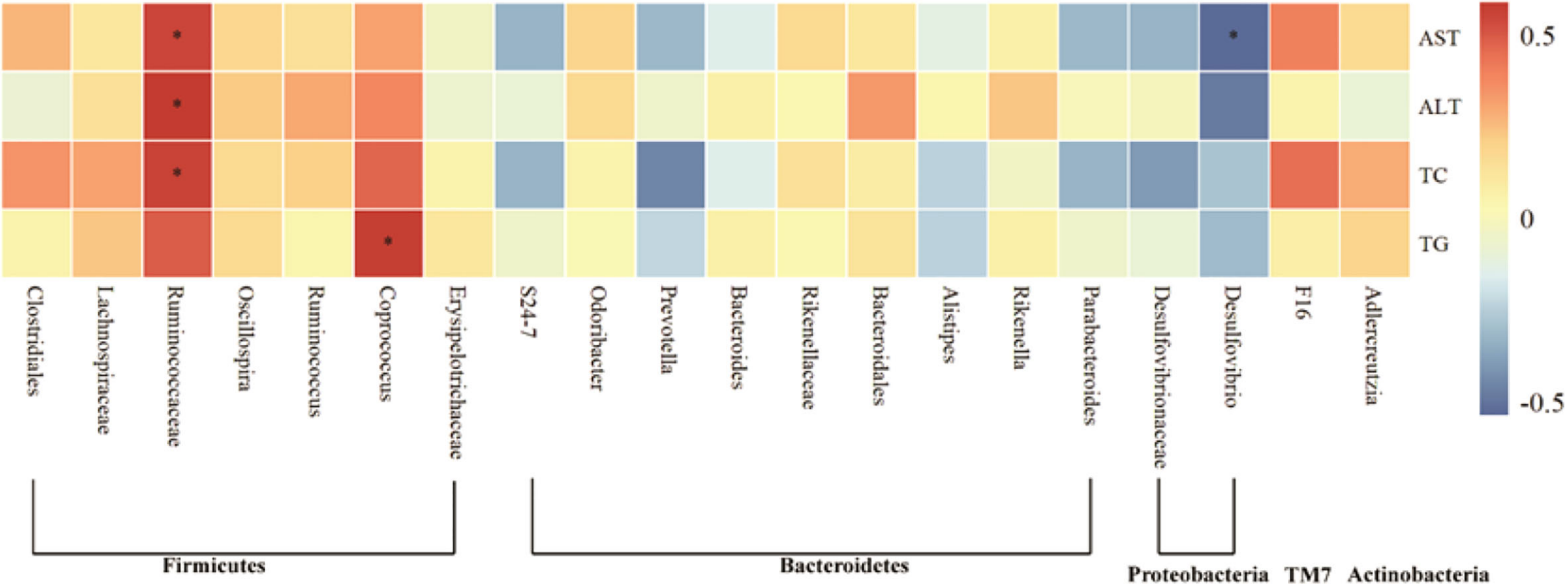
Heat map of the correlation of gut microbiota with indicators of liver injury in AFLD mice. Significant differences are indicated by asterisks, ^*^ *P* < 0.05.

To further determine the relationship between intestinal flora and AFLD induced by Chinese Baijiu exposure in mice, Spearman correlation analysis was performed in the current study. The relationship between liver function indicators including AST, ALT and blood lipid indicators including TC and TG and the top 20 genera in intestinal flora of mice was analyzed. As shown in **Figure 7**, The level of serum AST, ALT, TC and TG were positively correlated with *Firmicutes, TM7* and *Actinobacteria.* Among them, the serum AST, ALT and TC were positively correlated with *Ruminococcaceae* in *Firmicutes* (*P*<0.05). Moreover, the level of serum AST, ALT, TC and TG were negatively correlated with some genera in *Bacteroidetes*. Particularly, the serum AST was negatively correlated with *Desulfovibrio* in *Proteobacteria* (*P*<0.05).

## Discussion

The gut microbiota is the normal intestinal microbiota formed in the long-term evolution of the human body (20). They are distributed in the intestine with a certain regularity and form a defense barrier together with intestinal mucosa (21, 22). Many studies have shown that changes in the composition of gut microbiota affect host metabolism and are associated with diseases such as cirrhosis, diabetes, toxemia (23, 24). The gastrointestinal tract of the human body is the most important part of ethanol absorption and digestion, and long-term chronic drinking has adverse effects on the intestinal microecology. The accumulation of a large amount of ethanol in the intestinal tract will affect the intracellular signaling cascade and lead to the damage of various organs in the body. When the content of alcohol dehydrogenase in the body is low, the high concentration of ethanol cannot be metabolized, which will directly cause damage to the intestine and liver, including the bacterial translocation, the injury of intestinal barrier function, increased tissue inflammation, and the production of a large amount of endotoxin in the intestine (25). The liver and intestine are connected through the portal vein system, and a small amount of endotoxin enters the portal vein through the intestinal mucosa, which can maintain the hepatic reticuloendothelial system in an activated state. However, when the intestine is damaged, the imbalance of intestinal flora leads to a large amount of intestinal toxins entering the liver through the portal vein system, causing or aggravating liver injury (26, 27).

AST and ALT are important indicators to determine whether liver function is damaged, while TC and TG are the other two main indicators to measure liver lipid accumulation (28, 29). In the present study, the serum levels of AST, ALT, TC and TG in the administration group were significantly lower than those in the model group, indicating that the combination of HRP and APS can effectively regulate the liver function damage and lipid accumulation in AFLD mice. In addition, alpha diversity can reflect the diversity and richness of microbial communities *in vivo* (30, 31). 16S rRNA amplicon sequencing was used to decipher microbial diversity and abundance. Our study found that the Observed species, Chao1, Shannon and Faith’s PD index in the intestinal tract of the model group were remarkably decreased, indicating that alcohol consumption had a certain inhibitory effect on the richness and diversity of the intestinal flora in mice. After the polysaccharide intervention, the Observed species, Chao1, Shannon and Faith’s PD index in the intestinal tract of mice increased, and the HRP+APS group was close to the level of the blank group, which showed that combination of HRP and APS had a certain recovery effect on the intestinal microbial disturbance caused by alcohol consumption in mice.

By comparing the relative abundance changes of phylum levels in different groups of mice, we further analyzed how HRP+APS changed the intestinal microecology of AFLD mice. The change of relative abundance of *Fimicutes* and *Bacterodietes* is an important factor leading to the end-stage liver disease and other intestinal diseases. When the relative abundance of *Fimicutes* increases and the relative abundance of *Bacterodietes* decreases, inflammatory factors can be released and intestinal barrier is dysregulated. At the same time, the ratio of the relative abundance of *Fimicutes* and *Bacterodietes* can reflect the inflammatory state of samples. The results of this study showed that alcohol caused a significant increase in the F/B of the intestinal microbiota in mice, indicating that the intestinal microbiota in AFLD mice was dysregulated. After polysaccharide intervention, F/B decreased significantly, which was close to the blank group. It was speculated that HRP combined with APS was beneficial to the recovery of *Fimicutes* and *Bacterodietes* in AFLD mice. Both *Clostridiales* and *Lachnospiraceae* belong to the phylum *Fimicutes*. Among them, *Clostridiales* plays an important role in inducing and regulating immunity, mental diseases, puberty obesity and so on. At the same time, it plays an important role in intervening abnormal liver lipid metabolism and regulating fatty liver bile acid homeostasis, while *Ruminococcaceae* and *Lachnospiraceae* play a vital role in hepatic steatosis and lipid metabolism (32, 33). *Coprococcus* can actively ferment and decompose carbohydrates to produce such as butyric acid, acetic acid, formic acid, propionic acid, lactic acid, etc. Studies have shown that the change of *Coprococcus* is positively correlated with the changes of individual body weight, TC and TG (34). *S24-7* belongs to *Bacterodietes*, which can participate in the metabolism of human body. In the present study, correlation analysis indicated that the main changes of gut microbiota induced by alcohol treatment in mice were significantly positively correlated with hepatic steatosis. Furthermore, compared with the model group, HRP, APS and HRP + APS could increase the relative abundance of AFLD mice *S24-7* and reduce the relative abundance of *Lachnospiraceae*. Interestingly, APS was more likely to increase the relative abundance of *S24-7* in the gut of AFLD mice than HRP, and reduce the relative abundance of *Clostridiales* and *Lachnospiraceae* in the gut of AFLD mice. However, the combination of the two polysaccharides could reduce the effect of APS and make APS regulate the intestinal flora of AFLD mice more mildly.

## Conclusion

Excessive alcohol consumption can destroy the diversity and richness of intestinal microbiota in mice, and the combination of HRP and APS contribute to restore the diversity, relative abundance and community structure of intestinal mucosal bacteria to a certain extent. However, the effects of HRP and APS on intestinal microbiota of AFLD mice in dose and proportion need further study.

## Data availability statement

The original contributions presented in the study are publicly available. This data can be found here: https://github.com/lixiaoliang1894/Seabuckthorn-polysaccharide-combined-with-astragalus-polysaccharide-ameliorate-alcoholic-fatty-liver.git.

## Ethics statement

The animal study was reviewed and approved by Animal Ethics Committee of the Animal Experimental Center of Jiamusi University.

## Author contributions

HZ designed the study and revised the manuscript. LK, MS, JL, CS, and CL performed the experiments. YaW and XC analyzed the data. YuW and YZ supervised the work, and reviewed the manuscript. XL contributed to the study design and revised the manuscript. All authors reviewed the manuscript and approved the submitted version.

## Acknowledgments

This study was supported by the Foundation of Central Government Supports Local Universities (Grant No. 2019zyzcdf-01), Postdoctoral Research Special Fund of Heilongjiang Province (LBH-Q20185), North Medicine and Function Food Discipline of Heilongjiang Province (Grant No. 2018-TSXK-02), the Excellent Subject Team Project of Jiamusi University (Grant No. JDXKTD-2019005), and Scientific Research Support Project of Colleges and Universities in Hainan Province (Hnky2019ZD-24). The Technological Innovation Team Construction Project of Heilongjiang Provincial Department of Education (2021-KYYEF-0638).

## Conflict of interest

The authors declare that the research was conducted in the absence of any commercial or financial relationships that could be construed as a potential conflict of interest.

## Publisher’s note

All claims expressed in this article are solely those of the authors and do not necessarily represent those of their affiliated organizations, or those of the publisher, the editors and the reviewers. Any product that may be evaluated in this article, or claim that may be made by its manufacturer, is not guaranteed or endorsed by the publisher.

## Abbreviations

AFLD, Alcoholic fatty liver disease; TCM, traditional Chinese medicine; KM, Kunming; LPS, Lipopolysaccharide; ALT, Alanine aminotransferase; AST, Aspartate aminotransferase; TG, Triglyceride; TC, Total cholesterol; PCA, Principle component analysis; NMDS, Nonmetric multidimensional scaling; HRP, Hippophae rhamnoides polysaccharide; APS, Astragalus polysaccharide; H&E, Hematoxylin-eosin.
